# Cascaded Self-Supervision to Advance Cardiac MRI Segmentation in Low-Data Regimes

**DOI:** 10.3390/bioengineering12080872

**Published:** 2025-08-12

**Authors:** Martin Urschler, Elisabeth Rechberger, Franz Thaler, Darko Štern

**Affiliations:** 1 Institute for Medical Informatics, Statistics and Documentation, Medical University of Graz, 8036 Graz, Austria; 2 BioTechMed-Graz, 8010 Graz, Austria; 3 Institute of Computer Graphics and Vision, Graz University of Technology, 8010 Graz, Austria; 4 Gottfried Schatz Research Center, Medical Physics and Biophysics, Medical University of Graz, 8036 Graz, Austria

**Keywords:** semi-supervised learning, cardiac segmentation, self-training, student–teacher, pseudo-labeling

## Abstract

Deep learning has shown remarkable success in medical image analysis over the last decade; however, many contributions focused on supervised methods which learn exclusively from labeled training samples. Acquiring expert-level annotations in large quantities is time-consuming and costly, even more so in medical image segmentation, where annotations are required on a pixel level and often in 3D. As a result, available labeled training data and consequently performance is often limited. Frequently, however, additional unlabeled data are available and can be readily integrated into model training, paving the way for semi- or self-supervised learning (SSL). In this work, we investigate popular SSL strategies in more detail, namely Transformation Consistency, Student–Teacher and Pseudo-Labeling, as well as exhaustive combinations thereof. We comprehensively evaluate these methods on two 2D and 3D cardiac Magnetic Resonance datasets (ACDC, MMWHS) for which several different multi-compartment segmentation labels are available. To assess performance in limited dataset scenarios, different setups with a decreasing amount of patients in the labeled dataset are investigated. We identify cascaded Self-Supervision as the best methodology, where we propose to employ Pseudo-Labeling and a self-supervised cascaded Student–Teacher model simultaneously. Our evaluation shows that in all scenarios, all investigated SSL methods outperform the respective low-data supervised baseline as well as state-of-the-art self-supervised approaches. This is most prominent in the very-low-labeled data regime, where for our proposed method we demonstrate 10.17% and 6.72% improvement in Dice Similarity Coefficient (DSC) for ACDC and MMWHS, respectively, compared with the low-data supervised approach, as well as 2.47% and 7.64% DSC improvement, respectively, when compared with related work. Moreover, in most experiments, our proposed method is able to greatly decrease the performance gap when compared to the fully supervised scenario, where all available labeled samples are used. We conclude that it is always beneficial to incorporate unlabeled data in cardiac MRI segmentation whenever it is present.

## 1. Introduction

Cardiovascular Diseases (CVDs) are the most common non-communicable diseases globally [1]. Their prevalence increased from 5.1% in 1990 to 6.8% in 2019, with a rising trend in almost all low-income countries as well as in some high-income countries. Further, CVDs are also the leading cause of death globally, most prominently due to ischemic heart disease, ischemic strokes, and intracerebral haemorrhages [1]. Assessment of CVD risk can be performed using biochemical, genetic, or imaging-based biomarkers. Examples for imaging biomarkers relevant for CVD include dysfunctionality of the left ventricle, plaque burden, and tissue composition [2], which can be measured and monitored non-invasively using ultrasound, computed tomography (CT) or magnetic resonance imaging (MRI). Especially MRI is often used to assess volume and function of left and right ventricle, either statically or dynamically during the heartbeat [3]. To derive such biomarkers from MRI, a multi-compartment segmentation is required, allowing to compute functional metrics like ejection fraction, wall thickness, ventricle volumes, and myocardial mass that aid in diagnosing disease and in planning treatments [4].

Due to entirely manual expert segmentation being tedious, costly, and time-consuming, fully automated supervised methods have been proposed for multi-compartment segmentation, predominantly using deep neural networks like the U-Net or nnU-Net model [5,6]. To perform supervised learning, images and corresponding ground truth expert segmentation labels have to be available. These datasets need to be sufficiently large to learn generalizable models; however, providing large labeled datasets for training is still tedious and costly. For instance, manual annotation of 3D whole heart segmentation labels as performed for the MMWHS dataset [7] require 6–10 h of interaction time per MR or CT volume [8]. Thus, in medical imaging practice, the amount of labeled data is often limited.

Whereas labeled data might be scarce, unlabeled data are often abundantly available, which motivates the use of semi- and self-supervised learning (SSL) methods [9,10]. Such approaches learn from a combination of labeled and unlabeled data and promise improved efficacy and a reduction in labeling effort, both generally for medical image analysis and also specifically for cardiac multi-compartment segmentation.

In this work, we explore which types of semi- and self-supervised techniques achieve the best performance for segmenting cardiac images while keeping the number of labeled images as low as possible. Therefore, we comprehensively compare three baseline strategies that are widely used in the literature, i.e., Transformation Consistency (TC), Student–Teacher (ST), and Pseudo-Labeling (PL) [11]. Moreover, we also exhaustively combine the three aforementioned SSL strategies, thus forming individual cascaded multi-step approaches to investigate. We study all baseline and combined SSL methods by comparing them with a supervised baseline as well as several recent state-of-the-art SSL methods from the literature on two cardiac datasets involving both 2D and 3D images. Furthermore, to assess the performance of SSL when the size of labeled training data is decreasing, we construct different setups with smaller and smaller numbers of subjects in the labeled training set. Finally, built upon the findings of our SSL combinations, we propose a novel multi-step PL algorithm based on cascaded ST stages that most effectively profits from labeled and unlabeled data. To the best of our knowledge, this is the first PL method which uses an ST cascade with strong input data augmentations. Our main contributions are summarized as follows:

1. We propose a novel SSL strategy for cardiac MRI segmentation by performing multi-step pseudo-labeling based on cascaded ST stages.
2. We extensively evaluate different individual SSL strategies and their combinations and compare them with our proposed cascaded algorithm on 2D and 3D imaging data.
3. We assess the performance of all investigated strategies in a low-data regime by systematically reducing the size of labeled data and comparing with the fully supervised case as well as related work.

## 2. Related Work

The taxonomy of Yang et al. [11] proposes a distinction of SSL approaches into several categories, involving hybrid, graph-based, generative, consistency regularization-based, and Pseudo-Labeling-based methods. In our work, we focus on consistency regularization and Pseudo-Labeling approaches as well as hybrid combinations between those, since they do not pose as much computational overhead as generative synthesis methods [12,13]. Moreover, the considered methods are inductive as opposed to graph-based methods which, according to [11], are mostly transductive and thus not well applicable in our cardiac segmentation scenario, where future test images are generally not available in advance.

### 2.1. Consistency Regularization

Consistency regularization is based on the semi-supervised smoothness (cluster) assumption [9] and applies perturbations to the unlabeled data. Assuming the cluster assumption holds true, then perturbing data points within the same cluster should not change their predicted output semantically but solely perturb the output (segmentation labels) accordingly. The goal of consistency regularization is to minimize the discrepancy between the model output of an unlabeled data point and the accordingly perturbed model output of a transformed variant of the unlabeled data point. Transformations can be geometric (rotations, scaling, elastic deformation, etc.) as well as intensity-based (contrast variations, noise, blurring, etc.) [14]. Bortsova et al. proposed a Transformation Consistency approach for medical image analysis [15] using a Siamese network architecture with shared weights to segment chest X-ray images. Li et al. used transformations as well as dropout as a source of data perturbations to perform skin lesion segmentation of natural images [16]. Another line of work augments the input by masking parts of images and optionally combining them via mixing. Aiming to improve model robustness, DeVries et al. explored zero masking square parts of images for data augmentation in classification referred to as Cutout [17]. CutMix introduced by Yun et al. in [18] proposed to add random patches from the same images into the Cutout regions. French et al. then combined these two data augmentation techniques for consistency regularization in semantic segmentation [19].

An alternative manner to introduce consistency regularization is via multi-decoder consistency, where models have two or more branches which differ in their network architecture. Luo et al. implemented such an architecture simply by adding two additional distinct layers to a decoder, one for predicting segmentation outputs and the other to obtain a signed distance function representation of the output. They proposed their method as a dual task consistency (DTC) strategy, stating that the loss computes segmentations for labeled samples and additionally regresses signed distance functions for unlabeled samples [20]. Also, Wu et al. used two or three branches in their Mutual Consistency Network (MC-Net, MC-Net+) for left atrium segmentation with a common encoder and two or three separate decoders, where decoders differed in their upsampling strategies [21,22]. Three networks were also used by Huang et al., who proposed a main network and two auxiliary networks with varying skip connections in their decoders [23]. The outputs of the auxiliary models are passed through a sharpening function, thus serving immediately as a pseudo-label for the main model and the respective other auxiliary model.

In this study, we explore Transformation Consistency using strong data augmentation and make a comparison to various multi-decoder networks.

### 2.2. Student-Teacher

When combining the predictions of several neural networks by forming an ensemble, aggregated predictions tend to be more accurate compared to single networks. This idea inspired Laine and Aila for their temporal ensembling work [24] by reusing networks at different (i.e., previous) training epochs to produce predictions for unlabeled data. This strategy was further developed by Tarvainen and Valpola to form the Mean Teacher or Student–Teacher model [25]. Different from [24], in a Student–Teacher model, the student and the teacher model are both used at training time, where predictions of the teacher model are used as training targets of unlabeled data from which the student learns via an unsupervised loss.

Yu et al. introduced an uncertainty aware Mean Teacher for 3D left atrium segmentation [26] via several forward passes involving dropout. This lead to the student only learning from targets where the teacher exceeded a certain confidence threshold. Wang et al. extended this approach by using uncertainty to interpolate between student and teacher predictions for every voxel [27]. A variant of a certainty-driven consistency loss for Student–Teacher was proposed in [28] based on filtering targets from the teacher using top-k certain predictions. Huang et al. implemented Student–Teacher consistency regularization for neuron segmentation in electron microscopy volumes [29]. A proxy task is used to pre-train the encoder weights of the student network to reconstruct the original sample from perturbed versions, which helps the Student–Teacher network in making most effective use of unlabeled samples. Lei et al. also used the Mean Teacher model for medical segmentation and added two discriminators for an additional adversarial loss [30]. The goal of the first discriminator is to evaluate the quality of segmentation results, whereas the second discriminator differentiates between the perturbed and the original unlabeled samples. By combining Mean Teacher with Mixup augmentation [31], Basak et al. encouraged networks to linearly interpolate between training samples, leading to a very simple yet effective strategy [32].

In this work, we study different variants of the Student–Teacher paradigm and compare them with hybrid methods.

### 2.3. Pseudo-Labeling

There are two major directions in Pseudo-Labeling, self-training and deferring disagreement from different views or network models [11]. In self-training, an initially pre-trained network (e.g., using reconstruction error) is used to produce predictions of unlabeled data which are then treated as the reference annotation for training another network on the pooled training samples [33,34]. Contrarily, Bai et al. train their initial network in a supervised manner on their cardiac MR segmentation task directly [35]. Introducing prediction confidence via ensembles and re-generating pseudo-labels regularly after a certain number of training iterations is used in [36] in the context of lung infection segmentation. To form pseudo-labels via disagreement of different models, Li et al. suggest to use several permutations of the input (three by three equally sized tiles) and take the average of the predictions for each modified input as pseudo-label [37]. Xie et al. propose Noisy Student, a Student–Teacher-like framework, where a teacher network is trained in a supervised manner and generates pseudo-labels for unlabeled samples [38]. Another very recent state-of-the-art line of work combines self-training with contrastive learning, which aims to bring feature representations of similar labeled images closer together while pushing feature representations of dissimilar labeled images further apart. Chaitanya et al. propose Local Contrastive Loss with Pseudo-Labels (LCLPL) for semantic medical image segmentation, where they employ a contrastive loss on the pixel-level through a separate decoding branch using the ground truth for labeled data and pseudo-labels for unlabeled data [39]. Close to their work is the method proposed by Basak and Yin [40], who propose a patch-wise computation of contrastive loss instead of pixel-wise, which they call Pseudo-label Guided Contrastive Learning (PLGCL).

Pseudo-Labeling has been shown to be a promising SSL method recently [39,40]. Our evaluation demonstrates that combining the ideas of ST and PL can give a simple but very effective training scheme that is generally applicable for cardiac 2D and 3D MR segmentation and that achieves new state-of-the-art results.

## 3. Method

We design our study as a comprehensive evaluation of self-supervised strategies for cardiac MR image segmentation in low data regimes. Therefore, we investigate three methods: (i) Transformation Consistency, (ii) Student–Teacher without and with transformations, and (iii) Pseudo-Labeling. The following sections outline these methods and how we combine them, eventually leading to a novel cascaded self-supervised variant.

### 3.1. Transformation Consistency

**Figure 1:**
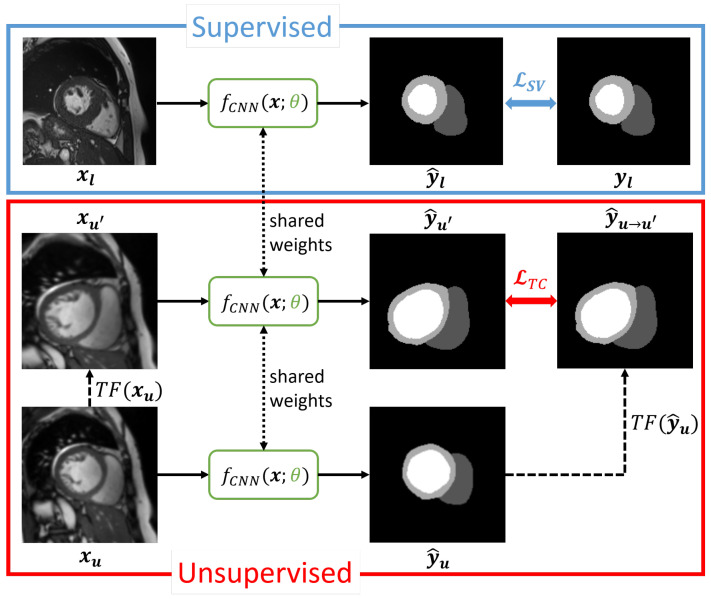
Transformation Consistency (TC) approach for self-supervised learning of cardiac multi-compartment segmentation. The supervised (SV) loss is accompanied by an unsupervised TC loss that penalizes deviations of differently transformed predictions. The segmentation CNN shares its weights between supervised and unsupervised components.

We build upon the Transformation Consistency work from [15], where every unlabeled sample $x_{u}\in X_{u}$ undergoes two different random transformations $TF_{1}\left(x_{u}\right)$ and $TF_{2}\left(x_{u}\right)$. The difference between the outputs for those two transformed samples forms the unsupervised loss component in their work. We modify their approach by solely using a single transformation $TF(x_{u})$ on unlabeled samples; see Figure 1 for an overview of the method. We use a set of potential geometric and intensity transformations inspired by [41], who apply the same transformations in the context of data augmentation for supervised multi-compartment cardiac segmentation. Each spatial transformation includes translation, rotation, scaling, and elastic deformation, with a uniformly sampled value within predefined ranges specifying the actual random transformation. For the intensity transformations, random shifting and scaling are chosen. Further details on the transformations can be found in Section 4.4.

In our method, two samples are randomly drawn during each training iteration. One sample $x_{l}$ is drawn from the set of labeled images $X_{l}$ for computing the supervised loss component $L_{SV}$, the other sample $x_{u}$ is drawn from the set of unlabeled images $X_{u}$ for computing the unsupervised loss component $L_{TC}$. For the unsupervised path, the sample $x_{u}$ is transformed to give $x_{u^{\prime }}=TF\left(x_{u}\right)$. The two variants $x_{u}$, $x_{u^{\prime }}$ are then forwarded through the single convolutional neural network (CNN) model f(x;θ), which produces corresponding predictions ${\hat{y}}_{u}=f\left(x_{u};\theta \right)$ and ${\hat{y}}_{u^{\prime }}=f\left(x_{u^{\prime }};\theta \right)$. The CNN shares its weights between supervised and unsupervised components. To align the two predictions, TF is applied to ${\hat{y}}_{u}$, resulting in the target for the unsupervised loss ${\hat{y}}_{u\to u^{\prime }}$. Ideally, ${\hat{y}}_{u^{\prime }}$ and ${\hat{y}}_{u\to u^{\prime }}$ should be identical. Thus, the unsupervised TC loss is computed as the difference between ${\hat{y}}_{u^{\prime }}$ and ${\hat{y}}_{u\to u^{\prime }}$, in the form of the mean squared error (MSE):

(1) $$L_{TC}=MSE\left({\hat{y}}_{u^{\prime }},{\hat{y}}_{u\to u^{\prime }}\right).$$

To compute the supervised loss component $L_{SV}$, we use a generalized Dice loss (GDL) [42], which compares predictions ${\hat{y}}_{l}=f\left(x_{l};\theta \right)$ with their available ground truth segmentation $y_{l}\in Y_{l}$. Both losses, supervised and unsupervised, are then combined to form the total SSL loss via a weighted sum, weighting the Transformation Consistency loss component with a factor λ:

(2) $$L_{SSL,TC}=L_{SV}+\lambda L_{TC}.$$

After the losses for the training are computed, the back-propagation of the gradient takes place. As ${\hat{y}}_{u\to u^{\prime }}=TF\left(f\left(x_{u};\theta \right)\right)$ serves as the unsupervised target for ${\hat{y}}_{u^{\prime }}$, ${\hat{y}}_{u\to u^{\prime }}$ is excluded from back-propagation by treating it like a constant to avoid gradient collapse as suggested in [43].

### 3.2. Student–Teacher

Motivated by the work of [25], we implement two ST models, where the teacher provides targets for the student model and in turn shares its knowledge via an exponential moving average (EMA).

In each ST training iteration, a labeled sample $x_{l}\in X_{l}$ and an unlabeled sample $x_{u}\in X_{u}$ are randomly drawn. The computation of the supervised loss is based on forwarding each labeled sample $x_{l}\in X_{l}$ through the student model only. Its output, ${\hat{y}}_{l}=f\left(x_{l};\theta_{S}\right))$ is compared with the ground truth segmentation $y_{l}$ via the GDL, same as for the Transformation Consistency method. To compute the unsupervised ST loss component $L_{ST}$, the teacher prediction ${\hat{y}}_{T}$ serves as a target for the prediction of the student ${\hat{y}}_{S}$. Both losses are combined to form the total SSL loss:

(3) $$L_{SSL,ST}=L_{SV}+\lambda L_{ST}.$$

After the back-propagation of the total SSL loss through the student network is performed, the weights of the student $\theta_{S}$ are updated. Conversely, the weights of the teacher network $\theta_{T}$ are not modified by back-propagation directly; instead, they are defined as the EMA of the student weights over all gradient updates and can be recursively defined as

(4) $$\theta_{T}^{i}=\alpha \theta_{T}^{i-1}+\left(1-\alpha \right)\theta_{S}^{i}.$$

We evaluate the ST loss component in two variants, leading to two distinct SSL methods.

#### 3.2.1. Student–Teacher Without Transformations

Here, the same input $x_{u}$ serves as input to the student model $f(x_{u};\theta_{S})$ and the teacher model $f(x_{u};\theta_{T})$. Both models use dropout during training for regularization. The MSE between the student predictions ${\hat{y}}_{S}=f\left(x_{u};\theta_{S}\right)$ and the target as predicted from the teacher ${\hat{y}}_{T}=f\left(x_{u};\theta_{T}\right)$ gives the unsupervised loss component $L_{ST}$:

(5) $$L_{ST}=MSE\left({\hat{y}}_{S},{\hat{y}}_{T}\right).$$

#### 3.2.2. Student–Teacher with Transformations

**Figure 2:**
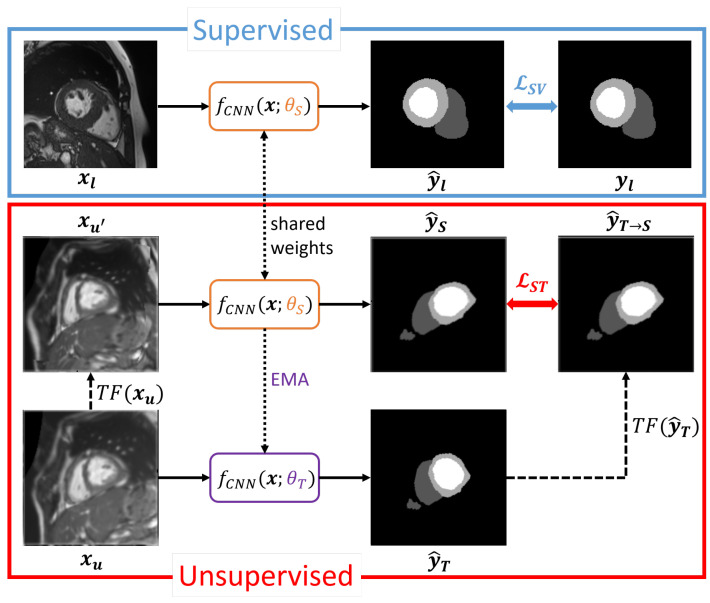
Student–Teacher approach with the use of Transformation Consistency for self-supervised learning. Different from pure TC, the teacher network weights are computed as an exponential moving average (EMA) of the student weights. Differently transformed predictions from student and teacher are penalized using the unsupervised ST loss component.

For the second ST variant (see Figure 2 for an illustration), Transformation Consistency as explained in Section 3.1 is used to additionally augment the unlabeled inputs. A random transformation TF is applied in each training iteration on a sample $x_{u}$. While the teacher $f(x;\theta_{T})$ receives the unmodified $x_{u}$ as input, $TF(x_{u})$ is forwarded to the student model $f(x;\theta_{S})$. The student outputs the prediction ${\hat{y}}_{S}$, whereas the teacher predicts ${\hat{y}}_{T}$. Same as for tTransformation Consistency, the predictions are aligned by applying TF on ${\hat{y}}_{T}$ to obtain ${\hat{y}}_{T\to S}=TF\left({\hat{y}}_{T}\right)$. The unsupervised loss $L_{ST}$ is then computed as MSE between ${\hat{y}}_{T\to S}$ and ${\hat{y}}_{S}$:

(6) $$L_{ST}=MSE\left({\hat{y}}_{S},{\hat{y}}_{T\to S}\right).$$

### 3.3. Self-Training via Pseudo-Labeling

**Figure 3:**
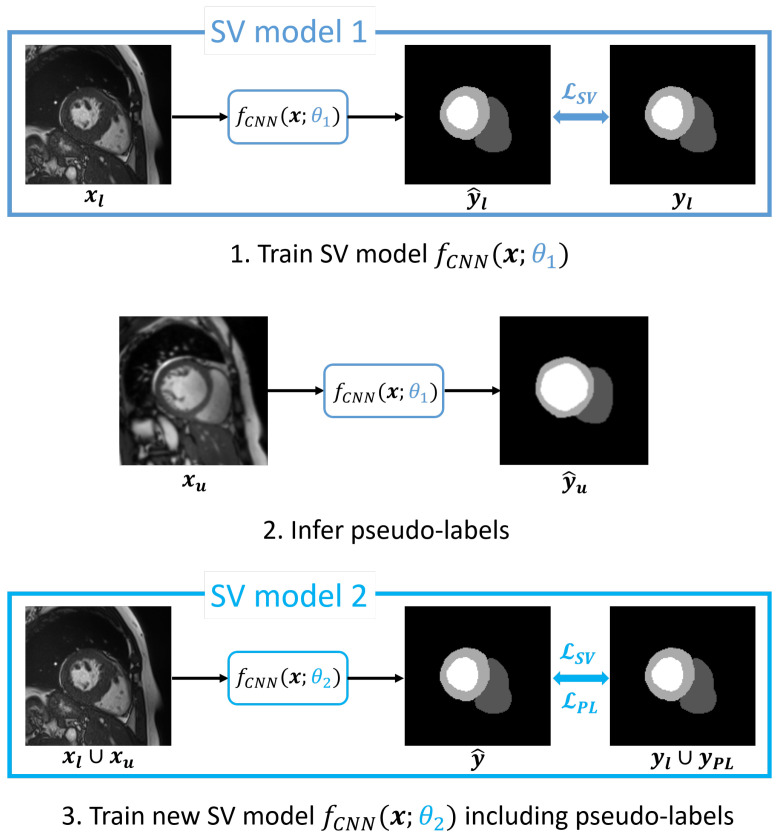
Traditional Pseudo-Labeling method based on two supervised training rounds. Model 1 trained in the first round is used in the second step to infer pseudo-labels for all unlabeled samples. In the second training round, the final prediction Model 2 is trained via a combination of supervised losses, which are computed using expert annotated ground truth segmentations for labeled data and the generated pseudo-labels for unlabeled data.

For Pseudo-Labeling variants, the training process consists of three consecutive stages. Firstly, the model $f(x;\theta_{1})$ is trained either based on the subset of supervised samples alone (see Figure 3) or by using one of the two previously discussed SSL strategies (TC or ST). Secondly, for every unlabeled sample $x_{u}\in X_{u}$, pseudo-labels are generated in an inference stage, leading to the set $Y_{PL}$. Finally, another model $f(x;\theta_{2})$ is trained from scratch with randomly initialized weights, but now in a purely supervised (SV) manner, with the union of $Y_{l}$ and $Y_{PL}$ as the set of target labels. For each iteration of the third stage, we randomly draw two samples, one sample $x_{l}$ from the labeled set $X_{l}$, the other sample $x_{u}$ from the originally unlabeled set $X_{u}$, both with their respective target label. This is performed to ensure that samples from the originally labeled set have sufficient influence during model training, as the number of pseudo-labeled samples is potentially much larger and their pseudo-label-based segmentations are expected to have a lower quality compared to samples for which actual ground truth segmentations are available. We note that we do not perform any filtering or selection of pseudo-labels but use all available pseudo-labels for $Y_{PL}$. The supervised loss in the final stage is composed of two different supervised losses, which are both implemented with a GDL. We separate the two losses to introduce a weighting factor for controlling the influence of the samples drawn with pseudo-label targets, leading to the total pseudo-labeling loss:

(7) $$L_{SSL,PL}=L_{SV}+\lambda L_{PL},$$

where $L_{SV}$ penalizes discrepancies between predictions ${\hat{y}}_{l}=f\left(x_{l};\theta_{2}\right)$ and targets $y_{l}\in Y_{l}$ and $L_{PL}$ penalizes discrepancies between ${\hat{y}}_{u}=f\left(x_{u};\theta_{2}\right)$ and targets $y_{PL}\in Y_{PL}$.

### 3.4. Cascaded Self-Supervision

**Figure 4:**
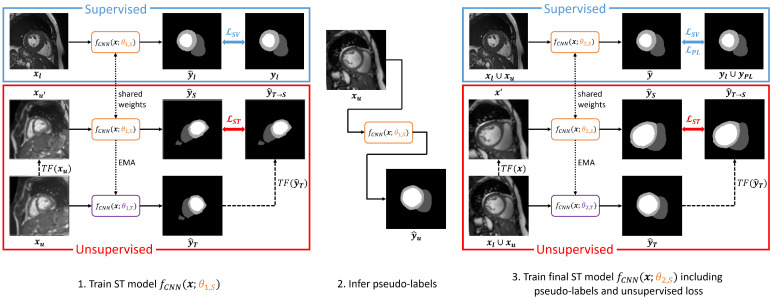
Proposed cascaded Self-Supervision method which employs Pseudo-Labeling and introduces a self-supervised cascaded Student–Teacher model, i.e., using self-training in the first and second training rounds (${\mathrm{ST}}_{\mathrm{TC}}{-\mathrm{PL}-\mathrm{ST}}_{\mathrm{TC}}$). Both ST models benefit from labeled and unlabeled samples during training. In addition, the second ST model is trained using pseudo-labels for the unsupervised set obtained through the first ST model. This results in two supervised and one unsupervised loss components.

Our main methodological contribution is to combine the idea of Student–Teacher with Pseudo-Labeling, thus creating a cascade of self-trained SSL approaches (see Figure 4). We hypothesize that such a cascade achieves a best of both worlds approach for segmentation, combining supervised and unsupervised components with as much training data as possible. Firstly, we train a model with Student–Teacher based on an unsupervised and a supervised set of samples, as described in Section 3.2. Secondly, we use the trained SSL model to infer pseudo-labels $Y_{PL}$ for all $x_{u}\in X_{u}$. We then combine the predictions $Y_{PL}$ with the ground truth labels $Y_{l}$, thus forming a label set $Y_{full}=Y_{l}\cup Y_{PL}$. Different from before, in the third stage, we train a new Student–Teacher model, where labeled samples $x_{l}$ are now drawn from $X_{full}=X_{l}\cup X_{u}$ due to the availability of pseudo-labels in $Y_{full}$. Samples $x_{u}$ are also drawn from $X_{full}$ but ignoring any ground truth labels. To balance labeled and unlabeled images such that pseudo-labeled images do not dominate during training, we draw one sample of each category per training iteration. Thus, we achieve a cascade of two semi-supervised Student–Teacher models, which makes optimal use of labeled, pseudo-labeled, and unlabeled samples simultaneously. The loss function in the third stage uses a weighted combination of GDL terms for the labeled ($L_{SV}$) and pseudo-labeled samples ($L_{PL}$), as well as a weighted MSE term for the unlabeled samples ($L_{ST}$):

(8) $$L_{SSL,cascade}=L_{SV}+\lambda_{1}L_{PL}+\lambda_{2}L_{ST}.$$

### 3.5. Datasets

**Figure 5:**
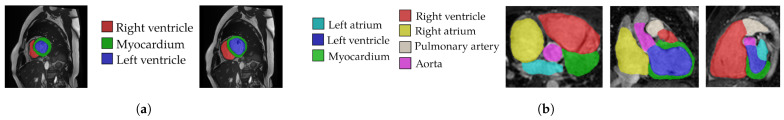
Datasets used for evaluating our self-training approaches in the cardiac MRI setting. (**a**) ACDC dataset [44] consisting of dynamically acquired 2D slices of the heart and providing a three-label annotation. (**b**) MMWHS dataset [7] consisting of 3D MR volumes with high spatial resolution and providing a seven-label annotation.

We use two cardiac segmentation datasets for evaluating our semi-supervised self-training methods. The ACDC dataset consists of 4D Cine MR images, which capture morphological changes of the heart during the heartbeat [44]. Three labels are available for this dataset, namely myocardium (MYO), right ventricle (RV), and left ventricle (LV); see Figure 5a. Data acquisition was synchronized with an ECG, using an SSFP sequence in short axis orientation. While the exact numbers vary, on average, each 4D sample in the dataset consists of about 26 time steps and thus around 26 3D volumes that represent the cardiac cycle. Ground truth segmentations by medical experts are only acquired for the systolic and diastolic phase of the cardiac cycle, while the 3D volumes of the remaining time steps remain unlabeled. The 3D volumes themselves capture roughly 10 2D short-axis slices on average that cover the heart from base to apex with a significant slice thickness of 5 to 10 mm, thus leading to severe anisotropy. In comparison, the spatial in-plane resolution ranges from 1.37 to 1.68 mm^2^. Furthermore, the 2D short-axis slices of the ACDC dataset are in some cases misaligned due to motion during data acquisition. Due to the large slice thickness as well as the misaligned slices, we use the ACDC dataset in 2D, which is the same setup as in related work. The whole dataset comprises image data from 150 patients, divided into five evenly sized subgroups: one healthy group and four with cardiac diseases (myocardial infarction, dilated and hypertrophic cardiomyopathy, and abnormal ventricle). Of these 150 patients, 100 are part of the training set, while the remaining 50 patients belong to the official testing dataset for which we did not have access to the ground truth annotations. Consequently, for training and testing of the evaluated methods, only the training set with 100 patients is used in cross-validation.

## 4. Experimental Setup

As a second dataset, we use the MR data from the MMWHS challenge held in conjunction with MICCAI 2017 [7]. Specifically, the dataset consists of 60 MR heart volumes in 3D, covering the region from the upper abdomen to the aortic arch. While the training set consists of 20 samples with ground truth labels, the test set encompasses 40 samples for which ground truth segmentations are not publicly available. The free-breathing MR volumes were acquired with a navigator-gated balanced SSFP sequence, with a nearly isotropic resolution of approximately (0.8−1)×(0.8−1)×(1−1.6) mm after the reconstruction. The segmentation labels include seven cardiac substructures, also shown in Figure 5b. These are the blood cavities, i.e., left ventricle (LV), right ventricle (RV), left atrium (LA) and right atrium (RA), the myocardium (MYO) of the left ventricle, the aorta (AO) starting from the aortic valve to the upper part of the atria, and the pulmonary artery (PA) including the pulmonary valve up to the bifurcation point.

### 4.1. Data Preprocessing

ACDC: Due to the anisotropic resolution of the ACDC dataset, we extract the 2D slices from each time step. With about 26 time steps and ten slices in the out-of-plane dimension on average, this results in around 260 2D images per patient. Two patients (IDs: 94, 88) were removed from the dataset, as their out-of-plane dimension was defined as superior to inferior, in contrast to the out-of-plane dimension for all other cases, which was defined as left to right. Additionally, slices were excluded from the dataset in case one or several of the labels covered less than 0.07% of the image in one of the two labeled time steps, since these slices introduced ambiguities due to inconsistencies in ground truth labeling of heart base and apex. In total, 25297 image slices of the ACDC dataset remained, of which approximately 7% (1670 slices) are labeled.

MMWHS and ACDC: To guarantee a common intensity range for the MR image intensities, robust normalization of MR images was performed by mapping the 95th percentile of intensity values per image to −1 and 1, respectively. Further, the intensity values for images and labels were preprocessed with Gaussian smoothing using a standard deviation of σ=1. Images and label masks are resampled to a size of 160×160 pixel with a spacing of 1×1 mm for ACDC slices and 96×96×96 voxel with 2×2×2 mm for MMWHS volumes to ensure a constant input size and resolution for the network. For resampling, we use linear interpolation for images and nearest neighbor interpolation for label masks.

### 4.2. Data Augmentation

To increase the diversity of the available images, we employ strong on-the-fly data augmentation during training using the framework of Payer et al. [41]. Both labeled and unlabeled samples are augmented using random spatial transformations. Sampled from a uniform distribution independently per dimensions, these include translation between [−20,20] pixels, rotation ranging from [−0.35,0.35] radians, and scaling with a factor ranging from [−20,20] pixels. Additionally, the unlabeled samples are transformed with randomized elastic deformations, with a maximum deformation value of 15 pixels and eight grid nodes for each dimension. Intensity transformations include random global shifting sampled from [−0.2,0.2] and global scaling ranging from [−0.4,0.4]. Due to the characteristic intensity distribution of MR images, we set intensities below −1 to −1.

### 4.3. Neural Network Architecture

We use a U-Net like architecture [5] to implement all our segmentation CNNs f(x;θ). The network consists of a contracting and an expanding path, as well as skip connections between them. The contracting and the expanding path consist of four blocks each. In both paths, one such block is composed of two convolution layers with zero-padding and 64 filter channels, as well as an intermediate dropout layer [45] with a dropout rate of 0.1 [46,47]. For the 3D inputs of the MMWHS dataset, we use a 3×3×3 kernel for convolution layers and a 3×3 kernel in the case of the 2D inputs of the ACDC dataset. Each convolution layer is followed by leaky ReLU as activation function with a slope of 0.1. After each block in the contracting path, we use average pooling for downsampling. Complementary, after each block in the expanding path, we employ linear upsampling, both with a factor of two. The skip connections concatenate intermediate features from the end of a block in the contracting path to the feature dimension of the matching level in the expanding path at the beginning of a block. Lastly, we employ softmax as the final activation function of the whole architecture to receive a pixel-wise probability distribution for each segmentation class before computing the losses.

### 4.4. Implementation Details

The weights of the convolution layers are initialized according to the method proposed in [48]. For each iteration during supervised training, we randomly draw one sample $x_{l}$ from the set of labeled samples $X_{l}$. When training semi-supervised methods, we additionally draw one sample $x_{u}$ from the set of unlabeled samples $X_{u}$ per iteration to balance the influence of labeled and unlabeled samples. The learning rate follows an exponential decay with a rate of 0.1. In the case of SV and TC training, the initial learning rate is $1\times 10^{-4}$, for ST and PL experiments the initial learning rate is set to $5\times 10^{-5}$. As optimizer, Adam [49] is selected with the decay rate parameters defined as $\beta_{1}=0.9$ and $\beta_{2}=0.999$. For ST, the EMA parameter α for the weight update of the teacher model is chosen as 0.999. For the consistency weighting factor λ for TC and ST, the factor 10 is chosen, whereas for PL training, the PL weighting factor is set to 1. For the cascaded ST method, $\lambda_{1}$ is set to 1 and $\lambda_{2}$ is set to 10. All weighting parameter choices are performed empirically, in a phase of initial experiments. The neural networks are trained for a total number of 180,000 iterations on the ACDC data and 40,000 iterations in the case of MMWHS. In all our self-training experiments, we train models for the first half of iterations in a supervised manner only. Only after this pre-training stage, the unsupervised learning paths are added to the training scheme, thus adding the influence of the unlabeled data. All parameters are selected after an initial empirical trial stage and according to prior experience in our group [41,46,50].

### 4.5. Evaluation Metrics

We evaluate the performance of all considered methods by computing the Dice Similarity Coefficient (DSC) in percent as well as the Average Symmetric Surface Distance (ASSD) in mm. All metrics are computed after linearly resampling the prediction $\hat{y}$ to its respective original spacing and dimension to allow a fair comparison. For multi-label segmentation, the DSC metric is defined as the average of the overlaps for all labels c∈C, i.e.,

(9) $$DSC\left(y,\hat{y}\right)=\frac{1}{\left|C\right|}\sum_{c\in C}\frac{2|y_{c}\cap {\hat{y}}_{c}|}{|y_{c}+{\hat{y}}_{c}|},$$

where y refers to the ground truth segmentation and $\hat{y}$ to the predicted segmentation. Complementary to the overlap-oriented DSC metric, the ASSD assesses the segmentation performance from a boundary-oriented perspective. The ASSD metric is defined as

(10) $$ASSD\left(y,\hat{y}\right)=\frac{1}{\left|C\right|}\sum_{c\in C}\frac{\sum_{y_{c}\in y_{c}}d\left(y_{c},{\hat{y}}_{c}\right)+\sum_{{\hat{y}}_{c}\in {\hat{y}}_{c}}d\left({\hat{y}}_{c},y_{c}\right)}{|y_{c}\left|+\right|{\hat{y}}_{c}|},$$

where d(·) is the Euclidean distance of a point *a* to the closest point *b* in a set of points b, i.e.,

(11) $$d\left(a,b\right)=\min_{b\in b}||a-b||.$$

For all our internal comparison experiments, scores are presented with their mean and standard deviation μ±σ and each experiment was repeated three times for every cross-validation fold. The standard deviation was first averaged over all fold repetitions for each sample and the final reported σ was computed over all cross-validation folds.

### 4.6. Self-Training Method Variants

In our comprehensive evaluation of self-training methods, we compare 10 different variants for both datasets, ACDC and MMWHS, with an increasing number of labeled samples in the supervised set, thus totaling four data regimes. There are four baseline methods, i.e., supervised (SV), Transformation Consistency (TC), Student–Teacher without TC (${\mathrm{ST}}_{\mathrm{noTC}}$), and Student–Teacher with TC (${\mathrm{ST}}_{\mathrm{TC}}$). Then, we derive three traditional supervised PL variants, where initial prediction models were trained either in a supervised manner (SV−PL−SV) or in a self-training manner (TC−PL−SV, ${\mathrm{ST}}_{\mathrm{TC}}-\mathrm{PL}-\mathrm{SV}$). Finally, we explore our proposed cascaded self-training combination ${\mathrm{ST}}_{\mathrm{TC}}{-\mathrm{PL}-\mathrm{ST}}_{\mathrm{TC}}$ and also investigate two ablation versions, i.e., ${\mathrm{ST}}_{\mathrm{noTC}}{-\mathrm{PL}-\mathrm{ST}}_{\mathrm{noTC}}$, as well as ${\mathrm{SV}-\mathrm{PL}-\mathrm{ST}}_{\mathrm{TC}}$.

### 4.7. Training Setup

Internal evaluation: To evaluate the effectiveness of our studied self-supervised methods, we created different data setups with an increasing size of patients with available ground truth annotations, but a constant size of unlabeled patients.

For ACDC, in each cross-validation fold, the image slices of 75 patients from the original training set were chosen as the per fold training dataset. The unlabeled set $X_{u}$ for ACDC is always composed of the image slices of all 75 patients from that fold. Contrarily, the labeled set $X_{l}$ consists either of 5 (7%), 15 (20%), 25 (33%) or all 75 (100%) labeled patients, giving our four supervised setups. Note that ground truth segmentations are only available for 2 out of the 25 time steps per patient, and consequently only the 2D slices of these two time steps are considered to be part of the labeled dataset (see Section 3.5). We use a four-fold cross-validation for each of the four setups; thus, there are always the labeled slices from 25 patients in the held out test set. To ensure that slices from one patient stay within training or test set of a fold, patient IDs were shuffled randomly across the splits as opposed to shuffling image slices.

For our internal evaluation of the MMWHS dataset, again, four different supervised setups with varying amounts of labeled patient samples were defined in a three-fold cross-validation, i.e., setups with 3 (20%), 5 (35%), 7 (50%), and 14 (100%) patient volumes out of the 20 potentially available annotated samples (6 were kept for respective test sets in the cross-validation). Thus, all setups contained 54 samples in total, which were used as unsupervised set $X_{u}$. Same as for ACDC, labeled samples were contained in the unsupervised set: $X_{l}\subseteq X_{u}$.

Comparison to recent related work: To also fairly compare the results of our proposed methods to the LCLPL work from [39], their evaluation setup was exactly reproduced for both MMWHS and ACDC. In contrast to our work, which employs the MMWHS data in 3D with size a 96×96×96 pixels and a physical resolution of 2×2×2 mm [39], image slices were extracted from the MMWHS volumes with a size of 160×160 pixels and a physical resolution of 1.5×1.5 mm. The fixed test set includes 20 patients for ACDC and 10 for MMWHS. For both ACDC and MMWHS datasets, three different supervised setups were constructed, containing the data of solely 1, 2, or 8 labeled patients each. This lead to percentages of 2%, 4%, or 16% for ACDC, as well as 10%, 20%, or 80% for MMWHS, respectively. The unlabeled sets comprised 10 patients for MMWHS and 52 for ACDC, respectively. The labeled patient images were also part of the unlabeled set: $X_{l}\subseteq X_{u}$. An entirely supervised baseline benchmark setup was also given, which comprised 78 ACDC patients or 10 MMWHS patients, i.e., (100%), respectively.

The second comparison was performed with PLGCL from Basak et al. [40], following the same setup from [51] for ACDC. In their test set, 20 patients were included. Another 70 different patients were used in each of the two different supervised setups, which included either 7 (10%) or 14 (20%) labeled patients. In their setup, these labeled patients were not part of the unsupervised set: $X_{l}\neg \subseteq X_{u}$. Also, an entirely supervised baseline benchmark setup was used, consisting of all 70 (100%) labeled patients. The unlabeled set consisted of 70 different patients from the dataset.

## 5. Results and Discussion

**Figure 6:**
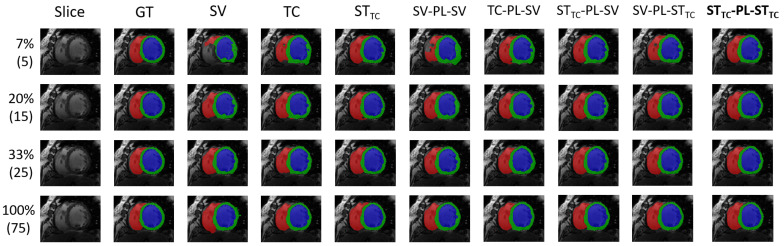
Exemplary qualitative results from ACDC using test subject ID 006. Rows refer to different percentages of labeled samples in the supervised set with the number of labeled patients given in brackets. While ${\mathrm{ST}}_{\mathrm{TC}}$ already achieves improvements over SV, even for solely 7% labeled images, the cascaded approaches, and especially ${\mathrm{ST}}_{\mathrm{TC}}{-\mathrm{PL}-\mathrm{ST}}_{\mathrm{TC}}$, give overall best results.

**Figure 7:**
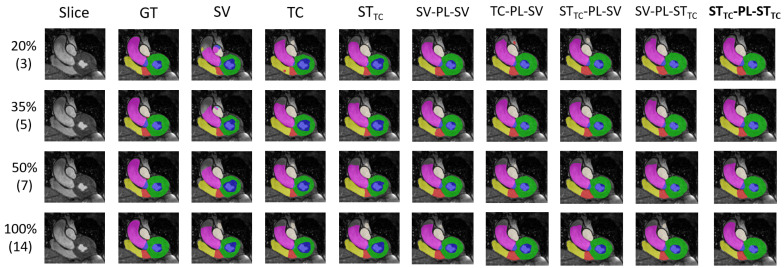
Exemplary qualitative results from MMWHS, using test subject ID 1001. Rows refer to different percentages of labeled samples in the supervised set with the number of labeled patients given in brackets. Again, ${\mathrm{ST}}_{\mathrm{TC}}{-\mathrm{PL}-\mathrm{ST}}_{\mathrm{TC}}$ delivers very promising predictions even in the low labeled data regime.

We provide the results on the ACDC dataset in Table 1 and results for the MMWHS are given in Table 2. Our most promising self-training SSL method ${\mathrm{ST}}_{\mathrm{TC}}{-\mathrm{PL}-\mathrm{ST}}_{\mathrm{TC}}$ as well as the corresponding baseline method ${\mathrm{ST}}_{\mathrm{TC}}$ are also compared to recent related methods. Our quantitative results when comparing to LCLPL from Chaitanya et al. [39] can be found in Table 3, whereas results when comparing to PLGCL [40] can be found in Table 4. Lastly, we also show some qualitative results for ACDC in Figure 6 as well as for MMWHS in Figure 7.

### 5.1. Internal Evaluation

Overall, the results of our evaluation shown in Table 1 and Table 2 demonstrate that using unlabeled data via any baseline self-training method improves segmentation results. This is the case for all scenarios, where a restricted number of labeled patients is used, and also for fully supervised (100%) scenarios, indicating that adding unlabeled data is never detrimental and can thus always be considered. Importantly, the performance gains of self-training compared with the entirely supervised variant become more prominent the smaller the size of the labeled set is, which highlights the effectiveness of SSL in the low-labeled data regime. We thus argue that the unsupervised losses can generally distill meaningful additional information from the unsupervised samples in cardiac MR segmentation. This aids in reducing tedious and costly labeling effort.

Out of the three baseline self-training methods, ${\mathrm{ST}}_{\mathrm{TC}}$ shows the largest performance increases over the supervised baseline, even giving overall best results in a few experiments. It seems to effectively combine the advantages of Transformation Consistency and Student–Teacher and is therefore studied in more detail in the various experiments when combined with pseudo-labeling methods. When introducing pseudo-labeling, additional performance gains can be observed, most strongly in the low-labeled data regime. While the pseudo-labeling baseline SV−PL−SV seems to have limitations due to the quality of initial pseudo-labels not being good enough, starting from ${\mathrm{ST}}_{\mathrm{TC}}$-based pseudo-labels it outperforms most simpler methods even when using a purely supervised training in the second round. However, in most experiments, supervised training in the second round can not fully compete with a cascaded self-training approach as proposed. We tested three cascaded Student-Teacher variants and found that the ${\mathrm{ST}}_{\mathrm{TC}}{-\mathrm{PL}-\mathrm{ST}}_{\mathrm{TC}}$ approach shows the most promising results in terms of performance gains for ACDC and MMWS across all studied metrics and all label percentage setups. Except for a few still close results, ${\mathrm{ST}}_{\mathrm{TC}}{-\mathrm{PL}-\mathrm{ST}}_{\mathrm{TC}}$ is either the best performing or second best performing method and shows low standard deviations among repetitions of the evaluations with different random seeds for training. Most notably, the boost between purely supervised and our proposed cascaded ST method in the lowest data regimes for ACDC and MMWHS is 10.17% and 6.72% in DSC, respectively. Our findings from the quantitative results are also confirmed when looking at predictions qualitatively. Both for ACDC (Figure 6) and MMWHS (Figure 7), the purely supervised approach (SV) shows segmentation errors in the low-labeled data regime. However, switching to ${\mathrm{ST}}_{\mathrm{TC}}$ gives great improvements, whereas including SV into Pseudo-Labeling is not always beneficial (see Columns 6 to 9, especially for ACDC). Qualitative results are consistently best when using our cascaded self-supervised approach ${\mathrm{ST}}_{\mathrm{TC}}{-\mathrm{PL}-\mathrm{ST}}_{\mathrm{TC}}$. We conclude from our extensive experiments that it is always beneficial to train a cascade of ST networks when unlabeled data are available in the cardiac MRI segmentation setting.

### 5.2. Comparison to Literature

Two recent works with state-of-the-art SSL methods, LCLPL [39] and PLGCL [40], were used to perform a comparison with our proposed ${\mathrm{ST}}_{\mathrm{TC}}{-\mathrm{PL}-\mathrm{ST}}_{\mathrm{TC}}$ method. Chaitanya et al. [39] evaluated several SSL methods on MMWHS and ACDC and also assessed the performance in the most challenging data regime evaluated in this work, where solely one patient was used in the labeled dataset. For both ACDC and MMWHS (see Table 3), they showed that when trained solely supervised, their reduced labeled data results are far from the corresponding supervised baseline, which is using 100% of available labeled data. LCLPL is able to bridge this gap using SSL in all scenarios and coming close to supervised baselines for as little as 16% or 20% labeled samples, respectively. However, both our compared methods, the baseline ${\mathrm{ST}}_{\mathrm{TC}}$ and the proposed ${\mathrm{ST}}_{\mathrm{TC}}{-\mathrm{PL}-\mathrm{ST}}_{\mathrm{TC}}$, outperform all related works drastically on both ACDC and MMWHS. Specifically, for 16% on ACDC and 20% on MMWHS, they perform on par with the upper bound supervised baseline, a behavior that was also seen in our comprehensive internal evaluation. In the most challenging 2% ACDC setting, we can see that our cascaded self-training, although outperforming all related work, is not competitive when compared with baseline Student–Teacher. We assume that when using solely one labeled patient in the first round, pseudo-labeling seems to overfit to this sample, limiting its overall performance. However, with as little as 4% labeled subjects, the proposed cascaded self-supervised approach is already beneficial with a 3.86% improvement in DSC. The behavior for MMWHS indicates that there is no clear winner between baseline Student–Teacher and the cascaded version. We argue that in this setup, the very low number of actually used unlabeled samples (10) is not sufficient to significantly benefit from. In comparison, our internal evaluation showed that using the remaining 40 unlabeled samples of MMWHS lead to consistently better cascaded self-training results. However, in the very-low-data regime setup, ${\mathrm{ST}}_{\mathrm{TC}}{-\mathrm{PL}-\mathrm{ST}}_{\mathrm{TC}}$ still outperforms the best related work by 7.64% DSC.

Our results in Table 4 demonstrate that our proposed method is also able to outperform the approaches in the ACDC-based evaluation setup that was used by Basak and Yin [40] to assess their PLGCL method. In the first reduced labeled patient setup, with seven subjects form the labeled set (10%), our ${\mathrm{ST}}_{\mathrm{TC}}{-\mathrm{PL}-\mathrm{ST}}_{\mathrm{TC}}$ method performs the best compared with all presented methods, including also LCLPL [39]. The difference in DSC to the second best PLGCL method is +2.47%. In the other reduced label setup, which includes 14 labeled subjects (20%), ${\mathrm{ST}}_{\mathrm{TC}}$ and ${\mathrm{ST}}_{\mathrm{TC}}{-\mathrm{PL}-\mathrm{ST}}_{\mathrm{TC}}$ show almost identical results and again surpass all other presented methods, with a DSC difference to PLGCL of +0.3%. Notably, the performance gap to the fully supervised baseline using a total of 70 patients again is nearly closed by our proposed cascaded self-training method in this experiment. We assume that the much larger number of unlabeled patients (70) is very beneficial in this experimental setup by [40], as opposed to the setup from [39].

### 5.3. Limitations

While our experiments are comprehensive for the 2D and 3D cardiac imaging domain, our strategies are supposedly more generally applicable. Thus, our conclusions are necessarily limited to this domain and an extension to other domains, e.g., abdominal organ segmentation, might be valuable next steps to follow up on. Moreover, we currently do not consider whether self-supervised training might help in generalizing within dynamical scenarios, e.g., when training a model for one cardiac timepoint and applying it to a different timepoint, making this another interesting direction for future work.

Methodologically, from our comparison to recent related work, which makes use of a contrastive loss component, we can see that our baseline and cascaded approaches do not require such a component to achieve state-of-the-art results. Nevertheless, due to contrastive losses being widely used in computer vision and medical image analysis applications recently, it may be beneficial to additionally incorporate such a loss term into our unsupervised component. We intend to study such a combination in future work.

## 6. Conclusions

In this work, we comprehensively evaluated semi- and self-supervised learning strategies in the context of cardiac MR image segmentation. By studying transformation consistency, Student–Teacher, Pseudo-Labeling, and combinations thereof, we found a novel, highly promising combination by cascading two Student–Teacher SSL training rounds within a Pseudo-Labeling workflow. Our experiments on the 2D ACDC and 3D MMWHS training setup with reduced labeled datasets revealed that given a set of unlabeled samples which is often abundantly available, it is always beneficial to train segmentation networks in a self-supervised manner. Moreover, we experimentally demonstrated that even in very-low-labeled data regimes, we can improve upon supervised low data baselines (10.17% and 6.72% improvement in DSC for ACDC and MMWHS, respectively) but also upon recent state-of-the-art SSL techniques that use more sophisticated training strategies like contrastive learning (2.47% and 7.64% DSC improvement, respectively). Finally, we also conclude that with our proposed cascaded Self-Supervision strategy, it is even possible to nearly close the performance gap to the fully supervised scenarios, where all available labeled samples are used during training.

## Figures and Tables

**Table 1:** Results from our internal evaluation comparing nine SSL methods and the supervised (SV) baseline on 2D ACDC dataset. Four different cross-validation setups were used for each percentage of patients in the labeled set, ranging from the lowest data regime of 7% up to 100%. Presented are the mean and standard deviation of DSC in % and ASSD in mm over three repetitions of the experiments. Best scores for mean and standard deviation are bold, second best scores are underlined.

Method	ACDC: Percentage of Patients in Labeled Set							
	7%		20%		33%		100%	
	DSC	ASSD	DSC	ASSD	DSC	ASSD	DSC	ASSD
	(%)↑	(mm)↓	(%)↑	(mm)↓	(%)↑	(mm)↓	(%)↑	(mm)↓
SV	78.26 ± 19.22	2.15 ± 2.35	86.57 ± 14.01	1.19 ± 1.40	87.14 ± 11.22	0.99 ± 1.05	89.65 ± 7.80	0.92 ± 1.06
TC	84.18 ± 12.67	1.54 ± 1.68	88.01 ± 10.23	1.11 ± 1.33	88.81 ± 9.34	1.02 ± 1.24	89.73 ± 7.43	0.92 ± 1.03
${\mathrm{ST}}_{\mathrm{noTC}}$	85.19 ± 12.54	1.26 ± 1.39	88.98 ± 9.64	0.91 ± 1.14	89.69 ± 8.69	0.84 ± 0.96	90.62 ± 7.24	0.75± 0.86
${\mathrm{ST}}_{\mathrm{TC}}$	86.90 ± 10.86	1.14 ± 1.20	89.48± 8.88	0.87±0.99	89.88± 8.54	**0.83**±**0.88**	**90.81**±6.88	**0.75**±**0.83**
SV−PL−SV	85.72 ± 11.24	1.39± 1.67	88.98 ± 8.65	1.00 ± 1.19	89.44 ± 8.34	0.92 ± 1.03	89.93 ± 7.29	0.86 ± 0.91
TC−PL−SV	86.55 ± 10.20	1.27 ± 1.41	88.89 ± 9.08	0.99 ± 1.09	89.43 ± 8.39	0.94 ± 1.16	89.90 ± 7.43	0.87 ± 0.94
${\mathrm{ST}}_{\mathrm{TC}}-\mathrm{PL}-\mathrm{SV}$	87.57± 9.77	1.15 ± 1.27	89.31 ± 8.67	0.95 ± 1.12	89.66 ± 8.07	0.90 ± 1.05	90.12 ± 7.30	0.86 ± 1.00
${\mathrm{SV}-\mathrm{PL}-\mathrm{ST}}_{\mathrm{TC}}$	86.56 ± 10.62	1.17 ± 1.36	89.37 ± 8.22	0.91 ± 1.02	89.83 ± **7.38**	0.86 ± 0.93	90.18 ± 6.99	0.81 ± 0.86
${\mathrm{ST}}_{\mathrm{noTC}}{-\mathrm{PL}-\mathrm{ST}}_{\mathrm{noTC}}$	87.10 ± 9.51	1.13±1.18	89.02 ± 8.74	0.95 ± 1.08	89.53 ± 7.76	0.90 ± 0.99	89.89 ± 7.32	0.84 ± 0.94
${\mathrm{ST}}_{\mathrm{TC}}{-\mathrm{PL}-\mathrm{ST}}_{\mathrm{TC}}$	**88.43**±**8.90**	**1.02**±**1.08**	**89.82**±**7.96**	**0.86**±**0.96**	**90.04**±7.43	0.85±0.93	90.49±**6.82**	0.79 ± 0.85

**Table 2:** Results from our internal evaluation comparing nine SSL methods and the supervised (SV) baseline on 3D MMWHS dataset. Three different cross-validation setups were used for each percentage of patients in the labeled set, ranging from the lowest data regime of 20% up to 100%. Presented are the mean and standard deviation of DSC in % and ASSD in mm over three repetitions of the experiments. Best scores for mean and standard deviation are bold, second best scores are underlined.

Method	MMWHS: Percentage of Patients in Labeled Set							
	20%		35%		50%		100%	
	DSC	ASSD	DSC	ASSD	DSC	ASSD	DSC	ASSD
	(%)↑	(mm)↓	(%)↑	(mm)↓	(%)↑	(mm)↓	(%)↑	(mm)↓
SV	81.44 ± 6.02	3.12 ± 1.78	85.04 ± 5.01	2.45 ± 1.84	85.97 ± 5.13	2.06 ± 1.38	87.71 ± 3.69	1.57 ± 0.90
TC	85.72 ± 3.20	1.65 ± 0.53	87.29 ± 2.97	1.44 ± 0.50	87.63 ± 3.35	1.42 ± 0.55	88.36 ± 3.07	1.30 ± 0.46
${\mathrm{ST}}_{\mathrm{noTC}}$	84.08 ± 4.03	2.00 ± 0.79	87.17 ± 3.24	1.51 ± 0.59	88.01 ± 3.27	1.40 ± 0.58	88.84 ± 2.83	1.22 ± 0.42
${\mathrm{ST}}_{\mathrm{TC}}$	86.14 ± 2.92	1.55 ± 0.42	87.56 ± 2.86	1.40 ± 0.48	88.21 ± 2.96	1.32 ± 0.46	88.69 ± 2.91	1.24 ± 0.43
SV−PL−SV	85.19 ± 4.75	2.08 ± 1.04	87.74 ± 3.20	1.41 ± 0.57	88.71 ± 3.45	1.27 ± 0.52	89.30 ± 2.82	1.18 ± 0.43
TC−PL−SV	87.58 ± **2.61**	1.36±0.40	88.50 ± 2.72	1.27±0.45	89.06 ± 2.87	1.20 ± 0.42	89.25 ± 3.04	1.19 ± 0.46
${\mathrm{ST}}_{\mathrm{TC}}-\mathrm{PL}-\mathrm{SV}$	87.68±2.72	1.36 ± 0.43	88.55±2.66	1.29 ± 0.47	89.17 ± 2.68	1.22 ± 0.42	89.42 ± 2.57	1.15 ± 0.38
${\mathrm{SV}-\mathrm{PL}-\mathrm{ST}}_{\mathrm{TC}}$	85.60 ± 4.73	2.02 ± 1.09	88.18 ± 3.17	1.32 ± 0.53	89.30± 2.94	1.15± 0.44	**90.04**±2.48	**1.05**±0.31
${\mathrm{ST}}_{\mathrm{noTC}}{-\mathrm{PL}-\mathrm{ST}}_{\mathrm{noTC}}$	85.67 ± 4.25	1.76 ± 0.80	87.54 ± 3.21	1.35 ± 0.49	89.12 ± 2.73	1.17 ± 0.41	89.76 ± 2.49	1.10 ± 0.35
${\mathrm{ST}}_{\mathrm{TC}}{-\mathrm{PL}-\mathrm{ST}}_{\mathrm{TC}}$	**88.16**± 3.02	**1.27**±**0.41**	**89.09**±**2.53**	**1.18**±**0.40**	**89.72**±**2.61**	**1.07**±**0.36**	90.00±**2.42**	1.06±**0.31**

**Table 3:** Results from comparison of our proposed method with Chaitanya et al. [39] on 2D ACDC and 3D MMWHS datasets, using their exact evaluation setup. Four percentages of patients in the labeled set were investigated. Mean DSC performance measures of related methods were taken from their publication, with the exception of the 100% supervised baseline result (*), which we reproduced. Best scores for mean and standard deviation are bold, second best scores are underlined.

Method	ACDC: DSC (%) ↑				MMWHS: DSC (%) ↑			
	Percentage of Labeled Patients				Percentage of Labeled Patients			
	2%	4%	16%	100%	10%	20%	80%	100%
Supervised from [39]	61.40	70.20	84.40	**91.20**	45.10	63.70	78.70	88.31 (*)
Noisy Student [38]	63.20	73.70	83.60	-	59.30	68.50	78.00	-
Mixup [31]	69.50	78.50	86.30	-	56.10	69.00	79.60	-
Self-Training [35]	69.00	74.90	86.00	-	56.30	69.10	80.10	-
LCLPL (inter) [39]	75.90	83.10	88.30	-	57.20	71.90	81.10	-
LCLPL (intra) [39]	76.10	84.50	88.10	-	59.90	72.10	80.30	-
${\mathrm{ST}}_{\mathrm{TC}}$ (ours)	**84.53**	87.90	89.66	-	64.63	**86.09**	**88.64**	-
${\mathrm{ST}}_{\mathrm{TC}}{-\mathrm{PL}-\mathrm{ST}}_{\mathrm{TC}}$ (ours)	78.00	**88.36**	**90.01**	-	**67.54**	85.81	88.56	-

**Table 4:** Results from comparison of our proposed method with Basak and Yin [40] on 2D ACDC dataset, using their exact evaluation setup. Three percentages of patients in the labeled set were investigated. Mean DSC performance measures of related methods were taken from their publication. We reproduced the supervised variants with our framework, as those were missing in [40]. Best scores for mean and standard deviation are bold, second best scores are underlined.

Method	ACDC: DSC (%) ↑		
	Percentage Labeled		
	10%	20%	100%
Supervised (ours)	86.54	88.63	91.92
Supervised from [40]	-	-	**92.30**
Double-UA [27]	83.30	-	-
DTC [20]	82.70	86.30	-
MC-Net [21]	86.30	87.80	-
MC-Net+ [22]	87.10	88.50	-
LCLPL [39]	88.10	90.50	-
PLGCL [40]	89.10	91.20	-
${\mathrm{ST}}_{\mathrm{TC}}$ (ours)	91.20	**91.52**	-
${\mathrm{ST}}_{\mathrm{TC}}{-\mathrm{PL}-\mathrm{ST}}_{\mathrm{TC}}$ (ours)	**91.57**	91.51	-

## Data Availability

The data sets presented in this study are available publicly.

## Abbreviations

The following abbreviations are used in this manuscript:

ACDC	Automated Cardiac Diagnosis Challenge
AO	Aorta
ASSD	Average Symmetric Surface Distance
CNN	Convolutional Neural Network
CT	Computed Tomography
CVD	Cardiovascular Diseases
DSC	Dice Similarity Coefficient
DTC	Dual Task Consistency
EMA	Exponential Moving Average
GDL	Generalized Dice Loss
LCLPL	Local Contrastive Loss with Pseudo-Labels
LA	Left Atrium
LV	Left Ventricle
MICCAI	Medical Image Computing and Computer Assisted Intervention
MMWHS	Multi-Modality Whole Heart Segmentation
MRI	Magnetic Resonance Imaging
MSE	Mean Squared Error
MYO	Myocardium
PA	Pulmonary Artery
PL	Pseudo-Labeling
PLGCL	Pseudo-Label Guided Contrastive Learning
RA	Right Atrium
RV	Right Ventricle
ST	Student–Teacher
SV	Supervised
TC	Transformation Consistency
SSL	Self-Supervised Learning
